# Enhanced Magnetism and Anomalous Hall Transport through Two-Dimensional Tungsten Disulfide Interfaces

**DOI:** 10.3390/nano13040771

**Published:** 2023-02-18

**Authors:** Chang-Ming Hung, Diem Thi-Xuan Dang, Amit Chanda, Derick Detellem, Noha Alzahrani, Nalaka Kapuruge, Yen T. H. Pham, Mingzu Liu, Da Zhou, Humberto R. Gutierrez, Darío A. Arena, Mauricio Terrones, Sarath Witanachchi, Lilia M. Woods, Hariharan Srikanth, Manh-Huong Phan

**Affiliations:** 1 Department of Physics, University of South Florida, Tampa, FL 33620, USA; 2 Department of Physics, The Pennsylvania State University, University Park, PA 16802, USA

**Keywords:** magnetic proximity effect, two-dimensional materials, iron oxide

## Abstract

The magnetic proximity effect (MPE) has recently been explored to manipulate interfacial properties of two-dimensional (2D) transition metal dichalcogenide (TMD)/ferromagnet heterostructures for use in spintronics and valleytronics. However, a full understanding of the MPE and its temperature and magnetic field evolution in these systems is lacking. In this study, the MPE has been probed in Pt/WS_2_/BPIO (biphase iron oxide, Fe_3_O_4_ and α-Fe_2_O_3_) heterostructures through a comprehensive investigation of their magnetic and transport properties using magnetometry, four-probe resistivity, and anomalous Hall effect (AHE) measurements. Density functional theory (DFT) calculations are performed to complement the experimental findings. We found that the presence of monolayer WS_2_ flakes reduces the magnetization of BPIO and hence the total magnetization of Pt/WS_2_/BPIO at *T* > ~120 K—the Verwey transition temperature of Fe_3_O_4_ (*T_V_*). However, an enhanced magnetization is achieved at *T* < *T_V_*. In the latter case, a comparative analysis of the transport properties of Pt/WS_2_/BPIO and Pt/BPIO from AHE measurements reveals ferromagnetic coupling at the WS_2_/BPIO interface. Our study forms the foundation for understanding MPE-mediated interfacial properties and paves a new pathway for designing 2D TMD/magnet heterostructures for applications in spintronics, opto-spincaloritronics, and valleytronics.

## 1. Introduction

Recently, two-dimensional transition metal dichalcogenides (2D-TMDs) have attracted a great deal of attention due to their extraordinary bandgap tunability, high optical sensitivity, strong spin orbit coupling, and low symmetry structure, making this a fascinating class of materials for spintronics, valleytronics, and spin caloritronics [1,2,3,4]. Spin degeneracy in momentum space at *K* and *K′* valley facilitates controllable spin polarization as detected by the helicity dependence of optical techniques such as magnetic circular dichroism [5] (MCD) and polarization resolved photoluminescence (PL). To enhance valley polarization, various approaches such as defect engineering [6], chemical doping [7], electrical doping [8], and TMD/FM (FM = ferro/ferrimagnet) heterostructures [5,9,10,11,12] have been exploited. Among these, TMD/FM heterostructures based on spin orbit coupling [13], charge transfer [9,14], and magnetic proximity effect [5,10] (MPE) are of topical interest. The MPE, an interfacial magnetic phenomenon due to the exchange coupling based on interfacial orbital hybridization [15]. Antiferromagnetic (AFM) exchange coupling was observed at the MoS_2_/yttrium iron garnet (YIG) interface by the MCD measurements at room temperature, where MoS_2_ flakes were transferred onto a YIG film via a wet transfer technique [5]. The ferromagnetic signal was detected in the MoS_2_ layer, induced by the ferrimagnetic YIG layer via the MPE. Density functional theory (DFT) calculations also reveal the charge transfer from YIG to MoS_2_ and the formation of n-type MoS_2_. However, Peng et al. reported the absence of MPE in the MoS_2_/YIG heterostructure [16]. This discrepancy may arise from the fact that the process of transferring 2D-TMDs to a magnetic substrate (via a wet or dry transfer process) affects the distance between the van der Waals material and the magnetic substrate and hence the strength of the orbital hybridization or the strength of the MPE. MoSe_2_ transferred onto La_0.7_Sr_0.3_MnO_3_ (LSMO) shows a reduction of the magnetization due to a destabilization of the double exchange coupling of Mn^3+^-O-Mn^4+^ at the MoSe_2_/LSMO interface by charge transfer [9]_._ On the other hand, Zhang et al. reported that the insertion of h-BN between MoSe_2_ and LSMO enhanced the valley splitting by the MPE [9]. These studies highlight that both charge transfer and the MPE are crucial for controlling the interfacial magnetic properties. A full understanding of such interfacial properties is the key to unlocking doors to the applications of spin-based nanodevices [17]. Interfacing YIG with graphene (Gr) can induce the MPE in the Gr layer as observed in magnetoresistance (MR) and spin pumping by the inverse Rashba effect [18]. Pulsed laser deposition grown MoS_2_/CoFe_2_O_4_ shows an enhanced MR effect when the number of MoS_2_ layers is reduced below 10 [19]. Since monolayer MoS_2_ exhibits relatively large spin orbit coupling and generates spin accumulation and spin injection, it may be responsible for the MR enhancement. The presence of an intermediate WSe_2_ monolayer (ML) has recently been shown to enhance the spin-charge conversion process, giving rise to the giant spin Seebeck effect in Pt/ML-WSe_2_/YIG [20]. First-principles calculations indicate the spin-Seebeck coefficient enhancement after the insertion of monolayer WSe_2_ between Fe and Pt layers in Pt/WSe_2_/Fe relative to Fe/Pt, due to the increases of the total density of state near the Fermi level and the magnetization [21]. In another study, first-principles calculations show the MPE when monolayer WS_2_ is placed on top of EuS [22]. Depending on the Eu- or S-surface termination, the interface with WS_2_ can exhibit ferromagnetic or antiferromagnetic ordering, which is also correlated with the distance between WS_2_ and EuS termination. Overall, previous works on different TMD/FM heterostructures mostly focused on magnetic insulators (e.g., YIG and EuS) that possess magnetic transition temperatures exceeding the range of the laboratory temperature (way above 300 K) [5,12,19,23]. It is of particular interest to study TMD/FM heterostructures in which the FM substrate undergoes magnetic/magneto-structural phase transitions in the measurable temperature range, which allows for another experimental degree of freedom to characterize the MPE and its temperature evolution via magnetic and transport measurements. In addition, it is essential to understand effects of different phases with different surface terminations of magnetic substrates on TMDs. In this context, we utilize a biphase iron oxide (BPIO = Fe_3_O_4_ + α-Fe_2_O_3_) film, which has recently been explored as an excellent model system for probing the impacts of phase coexistence on spin-thermo transport (via the spin Seebeck effect (SSE)) across heavy metal (HM)/FM interfaces [24].

Magnetite, Fe_3_O_4_, is a well-known half metallic ferrimagnetic material with a characteristic transition temperature called the Verwey transition, *T_V_* ~120 K. At this temperature, Fe_3_O_4_ shifts from a high temperature cubic halfmetallic phase to a low temperature monoclinic high resistance semiconducting/insulating phase due to the freezing of the Fe^3+^ and Fe^2+^ [25,26]. The half metallic nature above *T_V_* with high spin polarization makes it a potential candidate for a variety of spintronics applications [24,27,28,29]. The transport properties of pure Fe_3_O_4_ have been extensively studied [24,30,31,32]. In addition to Fe_3_O_4_, different forms of iron oxides exist, such as α-Fe_2_O_3_ [33,34], γ-Fe_2_O_3_ [35], ε-Fe_2_O_3_ [36], etc. Thus, the specific phase transitions associated with multiple phases of iron oxides in a single material may be utilized to probe detectable changes in iron-oxide/TMD heterostructures induced by the MPE [24,34]. The films prepared in this work consist of Fe_3_O_4_ and α-Fe_2_O_3_. While Fe_3_O_4_ is ferrimagnetic, α-Fe_2_O_3_ is an AFM material with Néel temperature (*T_N_*) above 900 K. Below the *T_N_*, α-Fe_2_O_3_ undergoes a first order spin-reorientation transition, also known as the Morin transition, *T_M_* ~250 K, with the Néel vector aligned along the basal plane and *c*-axis above and below the *T_M_*, respectively [37]. By exploiting changes in physical properties of Fe_3_O_4_ and α-Fe_2_O_3_ phases around their respective Verwey and Morin transitions, we show that the BPIO film is a model system for probing the interfacial magnetism in 2D-TMDs interfaced with a phase-tunable magnet. In this work, the interfacial phenomenon in Pt/ML-WS_2_/BPIO has been assessed by studying their magnetic properties, four-point resistivity measurements, and anomalous Hall effect (AHE) over a wide temperature range (10–300 K). The interfacial magnetism of the FM layer (BPIO) on the properties of the monolayer WS_2_ has also been studied by density functional theory (DFT), which fully supports the experimental findings. Our study provides new insights into the complex nature of magnetism at 2D-TMD/FM interfaces, enabling the design of 2D-TMD based heterojunctions with desirable properties for 2D van der Waals spintronics and valleytronics.

## 2. Materials and Methods

Monolayer WS_2_ flakes were grown on SiO_2_/Si using a chemical vapor deposition (CVD) technique. A prepared deionized water solution containing ammonium metatungstates ((NH_4_)_6_H_2_W_12_O_40_) and sodium cholate (C_24_H_39_NaO_5_) was spin-coated onto SiO_2_/Si substrate. The coated film was placed in a quartz tube with sulfur powder heated upstream. The quartz tube was heated in a furnace at 825 °C with Ar gas supplied. After the reaction process, the furnace was naturally cooled down to room temperature.

Biphase iron oxide (BPIO) films with a thickness of ~20 nm were grown on Si (100) substrates using the molecular beam epitaxy (MBE) technique. A 30-min preheating process was applied at 600 °C under high vacuum. Temperature was then cooled down to 400 °C for the film’s growth. Fe was evaporated with the rate of 0.2 Å/s under an oxygen pressure of 8.2 × 10^−6^ Torr.

To transfer WS_2_ flakes on top of BPIO, the wet transfer technique was employed. Poly(methyl methacrylate)) (PMMA) was first spin-coated on top of WS_2_ flakes. In order to reduce the solvent immersing time to remove PMMA in further steps, the spin-coated parameters were carefully adjusted to get proper PMMA thickness. Samples stood overnight for dehydration. Second, PMMA/WS_2_ flakes was immersed into potassium hydroxide (KOH) to detach WS_2_ from the Si substrate. After being successfully separated from the Si substrate, the PMMA/WS_2_ flakes were lifted off in DI water to clean several times. The PMMA/WS_2_ film was fished out by the BPIO substrate. Finally, PMMA was removed by acetone bath. To ensure the cleanness of the sample surface, extra isopropyl alcohol (IPA) was utilized after acetone bath and the surface was dried by N_2_ gas. Figure S1 shows the schematic of the wet transfer process.

The resulting WS_2_/BPIO and BPIO (reference) films were deposited platinum (Pt) of ~5 nm thickness by DC sputtering. The crystal phase structure of BPIO was characterized by a Bruker AXS powder X-ray diffractometer (XRD) with Cu Kα radiation. Raman spectrum was performed by a Horiba LabRAM HR Evolution Raman system with a 532 nm diode laser. Scanning transmission electron microscopy (STEM) image was collected in FEI Talos F220X microscope. Surface roughness was performed by VEECO Dimension 3100 Atomic Force Microscope (AFM). The magnetic properties of the films were characterized by the physical property measurement system (PPMS) from Quantum Design (QD), with a vibrating sample magnetometer (VSM) option. Resistivity measurements (four-point and anomalous Hall effect (AHE) measurements) were performed using the DC resistivity option of the PPMS within the temperature range 10 K ≤ *T* ≤ 300 K. The magnetic field was applied out of the film direction for the transport measurements.

The computational results are obtained based on DFT calculations using the Vienna Ab Initio Simulation Package (VASP) with the projector augmented wave method. The VASP code relies on periodic boundary conditions, and here all simulations are performed with the Perdew-Burke-Ernzerhof (PBE) generalized gradient approximation (GGA) [38,39,40]. Our recent study on the spin Hall magnetoresistance (SMR) of a BPIO film shows the presence of a dominant Fe_3_O_4_ phase on the surface of the film, and its effect on the spin transport in BPIO/Pt systems [41]. The effect of the α-Fe_2_O_3_ phase is found insignificant compared to the Fe_3_O_4_ phase. Therefore, our DFT calculations focus on the interface between Fe_3_O_4_ and WS_2_. The electronic shell structure has 8 valence electrons of Fe (3d^6^ 4s^2^), 6 valence electrons of O (2s^2^ 2p^4^), 6 valence electrons of W (6s^2^ 5d^4^), and 6 valence electrons of S (3s^2^ 3p^4^). The van der Waals interaction is also considered using the DFT-D3 method [42]. The Γ−point is used for Brillouin-zone integration and the electron wave function cut off energy is 450 eV. Electronic correlations are considered with Hubbard corrections for the Fe d-states, such that U=3.8 eV and J=0.0 eV for the cubic lattice, and U=4.50 eV and J=0.89 eV for the monoclinic lattice. Such values have been used by others when calculating electronic structure properties of Fe_3_O_4_ [43,44]. To model the Fe_3_O_4_/WS_2_ systems, we construct a superlattice structure with a slab of (2×2) cubic Fe_3_O_4_ (110) (72 Fe and 96 O atoms) and a $\left(3\sqrt{2}\times 4\sqrt{2}\right)$ WS_2_ monolayer (24 W and 48 S atoms) on top. We also construct a superlattice structure with a slab of Cc symmetry of the (2×2) monoclinic Fe_3_O_4_ (110) (72 Fe and 96 O atoms) and a $\left(3\sqrt{2}\times 5\sqrt{2}\right)$ WS_2_ monolayer (30 W and 60 S atoms) on top. The thickness of the Fe_3_O_4_ layer is 7.5 Å for the cubic, and 5.4 Å for the monoclinic. A vacuum layer of 15 Å separates the periodically repeating slabs. The structural optimization for all Fe_3_O_4_ configurations is performed by allowing full relaxation of the top two atomic layers while the atoms of the remaining four layers are kept at their bulk positions. Such a procedure has been utilized by others to simulate the role of the surface termination in the properties of magnetite [44,45]. The structural relaxation is carried out using the conjugate-gradient algorithm until the Hellmann–Feynman force on each atom is less than 0.01 eV/Å, and the total energy is less than 10^−5^ eV. To simulate the ferromagnetic properties of Fe_3_O_4_, the octahedral and tetrahedral Fe atoms have opposite spin orientations.

## 3. Results

### 3.1. Material’s Characterization

**Figure 1:**
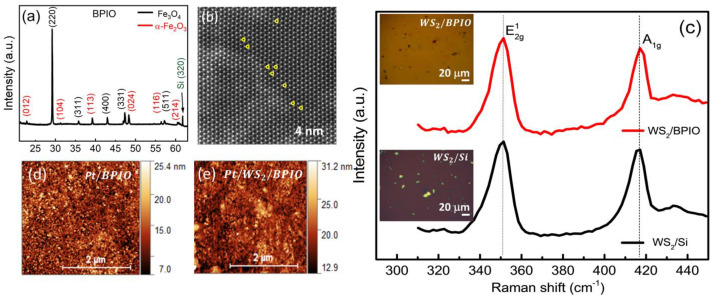
(**a**) X−ray diffraction (XRD) pattern for the 20 nm thick BPIO film. (**b**) WS_2_ flakes under high resolution scanning transmission electron microscopy (STEM). The yellow circles indicate S vacancies in WS_2_. (**c**) Raman spectra for WS_2_ flakes before (WS_2_/Si) and after (WS_2_/BPIO) the wet transfer process. Atomic force microscopy (AFM) was performed after Pt deposition for (**d**) Pt/BPIO and (**e**) Pt/WS_2_/BPIO. Insets of (**b**) indicate the optical images of WS_2_ flakes on Si (before the transfer process) and BPIO (after the transfer process), respectively.

To study the influence of the insertion of TMD monolayers on the physical properties of the HM/FM heterostructure, Pt/ML-WS_2_/BPIO and Pt/BPIO (the reference sample) were prepared (as detailed in Figure S1). WS_2_ flakes (monolayers) were wet transferred on top of a 20 nm thick BPIO film. Figure 1a represents the X-ray diffraction (XRD) pattern of our BPIO (20 nm)/Si film, confirming the coexistence of Fe_3_O_4_ and α-Fe_2_O_3_ phases. In addition, from the XRD data, the average crystallite size of Fe_3_O_4_ and α-Fe_2_O_3_ are estimated to be 115 nm and 55 nm, respectively, based on the Scherrer equation [46]. The coexistence of Fe_3_O_4_ and α-Fe_2_O_3_ phases is also confirmed by Raman spectroscopy as reported in our previous work [24]. High resolution scanning transmission electron microscopy (STEM) confirms the quality of the WS_2_ structure with some sulfur vacancies as seen in Figure 1b. The optical images in insets of Figure 1c show the WS_2_ flakes before and after transferring to the BPIO film. In addition, an estimation of WS_2_ flakes surface coverage is around 10.7%. The Raman spectra show two modes, $E_{2g}^{1}$ (351 cm^−1^) and *A*_1*g*_ (417 cm^−1^), of the WS_2_ flakes before (WS_2_ flakes/Si) and after transferring (WS_2_ flakes/BPIO), indicating a successful transfer process. A small bump (433 cm^−1^) next to *A*_1*g*_ peak appears to occur due to the presence of S vacancies in WS_2_ [47], which is evident in the STEM image (Figure 1b). A 5 nm layer of Pt was deposited on both WS_2_/BPIO and BPIO films, forming Pt/WS_2_/BPIO and Pt/BPIO heterostructures. The surface topologies of the Pt/WS_2_/BPIO and Pt/BPIO films were characterized by atomic force microscopy (AFM), as shown in Figure 1d,e. The average surface roughness of Pt/WS_2_/BPIO and Pt/BPIO are determined to be ~3.388 nm and ~3.377 nm, respectively, indicating similar surface characteristics for both films.

### 3.2. Magnetic Properties

**Figure 2:**
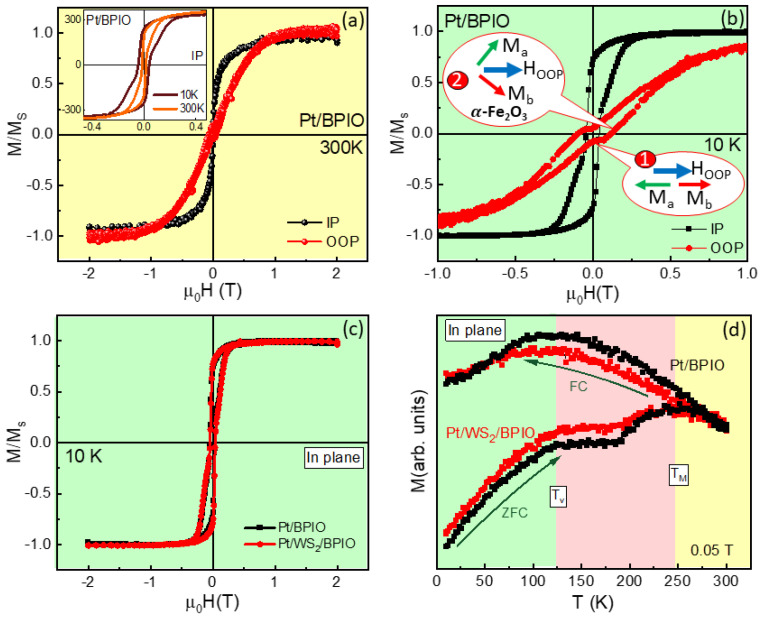
M−H curves are measured for (**a**) Pt/BPIO at 300 K in IP and OOP directions and (**b**) Pt/BPIO under low field at 10K in the IP direction. (**c**) IP M−H curves for Pt/BPIO and Pt/WS_2_/BPIO at 10 K. (**d**) M-T curves for both Pt/BPIO and Pt/WS_2_/BPIO for the IP direction. Inset of (**a**) has Pt/BPIO measured at 10 and 300 K for IP magnetic field. Inset of (**b**) indicates the α−Fe_2_O_3_ spin flop transition behaviors. M_a_ (green) and M_b_ (red) are two sublattices for α−Fe_2_O_3._ OOP magnetic field is noted as blue arrow.

The magnetic properties of the Pt/WS_2_/BPIO and Pt/BPIO films were then characterized using the vibrating sample magnetometer (VSM) equipped within a physical property measurement system (PPMS) from Quantum Design. Magnetization versus magnetic field (M-H) measurements were conducted on the Pt/BPIO film for both in-plane (IP) and out-of-plane (OOP) field directions. Figure 2a shows that BPIO possesses an IP easy axis at room temperature. As shown in the inset of Figure 2a, the IP M-H isotherms at 10 and 300 K clearly exhibit non-monotonic curves indicating bi-phase structures, as detected by the XRD. In addition, the coercive field (*H_C_)* is significantly enhanced as the temperature is lowered from 300 K to 10 K. The zoomed-in M-H curves for both IP and OOP M-H loops measured at 10 K are plotted in Figure 2b. Interestingly, a spin flop-like transition can be observed in the OOP M-H curve, likely due to the low field spin flop transition of the AFM α-Fe_2_O_3_ phase [37]. Since Fe_3_O_4_ is the dominant phase in the BPIO film, the spin flop-like behavior starts at an even lower field region, as compared to pure α-Fe_2_O_3_. When the magnetic field approaches zero, a plateau-like feature is observed, which can be attributed to the effect of the applied magnetic field which is insufficient to align the spin sublattices a and b in the α-Fe_2_O_3_ phase, and these two spin sublattices of α-Fe_2_O_3_ are anti-parallel to each other, as illustrated in inset #1 of Figure 2b. A gradual increase of the magnetic field reveals that the spin flop-like behavior occurs when the two sublattices rotate towards the magnetic field’s direction but do not fully align with it (inset #2 of Figure 2b). Because this spin flop-like behavior only occurs in the OOP measurement, it is reasonable to infer that the spin sublattice of α-Fe_2_O_3_ mostly lies in the OOP direction, and the Néel vector is in the IP direction. In Figure 2c, we compare the IP M-H curves for Pt/BPIO and Pt/WS_2_/BPIO at 10 K. The insertion of WS_2_ does not significantly alter the shape of the M-H hysteresis nor the saturation magnetization (*M_S_*). This can be reconciled to the fact that the induced magnetic moment in the WS_2_ layer is not detectable by the PPMS, which is also consistent with the small value of M obtained from DFT calculations. Figure 2c also indicates that the solvent clean process did not damage the BPIO surface and alter the nature of two phases after the wet transfer. Figure 2d shows zero field cooled (ZFC) and field cooled (FC) magnetization versus temperature (M-T) curves for both the films measured in a magnetic field of 0.05 T for the IP field direction. Upon cooling from room temperature, the ZFC M(T) first increases and shows a broad maximum around *T_M_* ~250 K and drops gradually down to 200 K. As the temperature goes down further, the ZFC *M(T)* shows a shoulder-like trend, and M starts dropping significantly around *T_V_* ~120 K toward the lowest temperature. The feature around *T_M_* ~250 K is associated with the Morin transition of the AFM α-Fe_2_O_3_ phase, while the feature at the *T_V_* originates from the Verwey transition of the Fe_3_O_4_ phase [24]. The relevant transition temperature regions have been highlighted below the *T_V_* (green), above the *T_V_* and below the *T_M_* (pink), and, above the *T_M_* (yellow). Overall, our XRD, Raman, and magnetometry data consistently show the coexistence of Fe_3_O_4_ and α-Fe_2_O_3_ phases in the BPIO film, and this feature is preserved in Pt/WS_2_/BPIO.

### 3.3. First Principles Calculations

**Figure 3:**
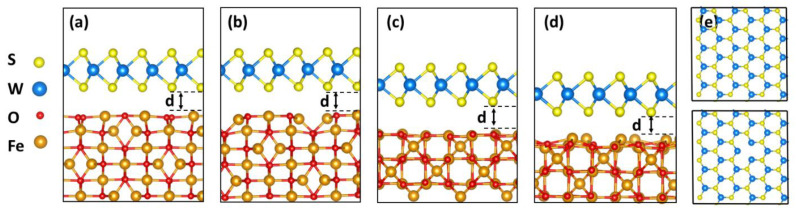
Side view of the simulated Fe_3_O_4_ (110)/WS_2_ heterostructures: (**a**) B-terminated cubic Fe_3_O_4_; (**b**) AB-terminated cubic Fe_3_O_4_; (**c**) B-terminated monoclinic Fe_3_O_4_; (**d**) A-terminated monoclinic Fe_3_O_4_. (**e**) Top view of WS_2_ and WS_2_ (V_S-S_) with two adjacent S vacancies.

Given that the Fe_3_O_4_ phase is predominant in the samples, we further investigate from first principles WS_2_/Fe_3_O_4_ heterostructures as a prototype of the magnetic interface properties of our experiments. The calculations are performed within DFT as implemented in the VASP code and relevant details are given in Methods. Despite the computational limitations due to the large number of atoms and the incommensurate lattices of the different components of the experimental heterostructures, we simulate several WS_2_/Fe_3_O_4_ heterostructures to discern between the low and high temperature phases of the magnetite. The supercells are constructed (details in Methods) by taking layers for the cubic and monoclinic phases with two types of (110) surfaces depending on the location of the surface Fe atoms. Given the polycrystalline nature of the presently studied BPIO film, different surface terminations of Fe_3_O_4_ are considered in our DFT calculations. These are given in Figure 3, where WS_2_ is placed above a B- and AB-terminated cubic Fe_3_O_4_ (Figure 3a,b, respectively), and B- and A-terminated monoclinic Fe_3_O_4_ (Figure 3c,d, respectively). In addition, pristine and defective WS_2_ monolayers containing ∼5% S vacancies are also considered. The defective monolayer is obtained by removing two adjacent S atoms, as shown in Figure 3e. The magnetite contains six atomic layers (see Figure 3), and, during the simulations, the top two layers are allowed to relax while the remaining four are kept fixed at their bulk positions. Such a procedure enables capturing the unique role of the magnetite surface and its properties, as shown by others [44,45]. Due to the incommensurate lattices of WS_2_ and Fe_3_O_4_, the construction of the supercells and subsequent relaxation introduce slight strain, which does not exceed 5% for each component.

Results from the calculations for the interlayer distance, binding energy, and magnetic moments for the considered heterostructures are summarized in Table 1. We find that the interlayer distances d are smaller than typical van der Waals separations, which are usually ≥3 Å. On the other hand, the binding energies between WS_2_ and Fe_3_O_4_ $E_{b}∼\left(-20,-35\right)$ meV are consistent with weaker interactions indicative of van der Waals coupling [48]. The calculated average magnetic moments of the two inequivalent Fe (A and B) and the O atoms further show that the direction of the atomic spin polarization is preserved regardless of the WS_2_ presence. Not only that, the magnitude of $m_{Fe}{}_{A}$, $m_{Fe}{}_{B}$, and $m_{O}$ are not changed significantly for most considered heterostructures. This is a consequence of the rather small magnetization of WS_2_ above the magnetite, consistent with the experimental observations (Figure 2). This small magnetic proximity effect is not influenced significantly by the type of Fe_3_O_4_ surface termination, nor the presence of defects in the WS_2_ monolayer. The average magnetization of the WS_2_/Fe_3_O_4_ heterostructures is practically the same, with the exception of the A-terminated monoclinic magnetite layer for which an enhancement of M is found when compared to the standalone Fe_3_O_4_. It is interesting to note the increase in M cannot be attributed to the proximity magnetization of WS_2_ (since $M_{WS_{2}}$ is insignificant), but it has a chemical origin due to orbital hybridization. These results show that the magnetic properties of the heterostructures are mainly determined by Fe_3_O_4_. The secondary role of WS_2_ is also in agreement with our measurements showing that the TMD does not significantly affect the magnetization properties of the heterostructure, as discussed above (also, see Figure 2).

### 3.4. Four-Probe Measurement

**Figure 4:**
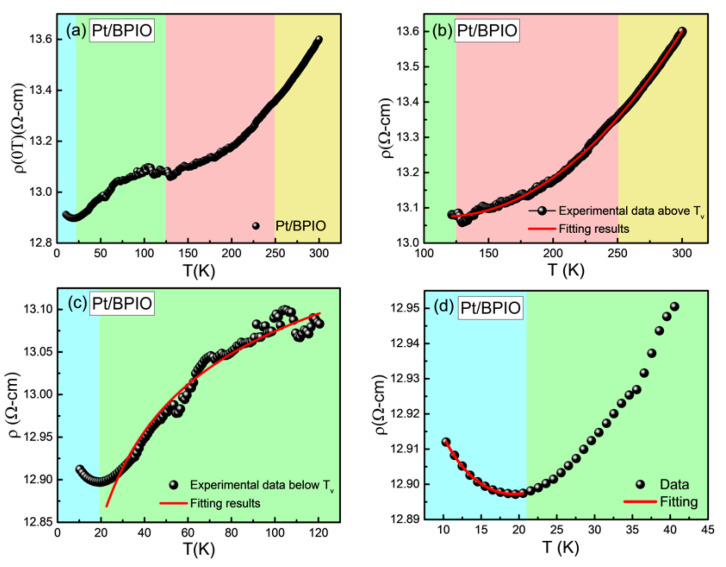
Zero field resistivity measured at temperatures between 10 and 300 K for (**a**) Pt/BPIO. Curves fitted for temperatures (**b**) above and (**c**) below the *T_V_* in Pt/BPIO and (**d**) the minimum resistivity behavior noted below 20 K.

To understand the effect of WS_2_ on the transport properties of the Pt/BPIO film, the temperature dependence of electrical resistivity ρ(T) was measured using a standard four-probe (FP) measurement technique in the absence of external magnetic field, as shown in Figure 4a. Interestingly, ρ(T) does not exhibit sharp changes around the two transition temperatures observed in our BPIO system, namely the Morin transition associated with the α-Fe_2_O_3_ phase and the Verwey transition associated with the Fe_3_O_4_ phase. This can be understood because the BPIO film is not epitaxial. The resistivity of this film around the *T_V_* does not increase as significantly as that grown on MgO [49]. The Verwey transition temperature can still be observed in the ρ(T) measurement. However, we cannot directly determine the transition temperature from the derivative of ρ(T) and broad maximum around the transition regions. This is because the BPIO system contains a biphase structure which could influence each other and broaden/shift the transition temperature. Above the *T_V_*, resistivity gradually increases without any significant slope change (Figure 4), as observed in the M-T curve (Figure 2). The absence of *T_M_* in ρ(T) is due to the small volume fraction of the α-Fe_2_O_3_ phase present in the BPIO film, and the consideration that the α-Fe_2_O_3_ phase is insulating throughout the measured temperature range. Chanda et al. [24] also found the absence of *T_M_* in the FP measurements. On the other hand, an additional transition temperature appears in the ρ(T) curve at a low temperature. To ascertain the origin of this low temperature behavior, different mechanisms for charge carrier conduction are discussed. Above the *T_V_*, while Fe_3_O_4_ has a half-metallic characteristic, we have a bi-phase structure for the presently studied sample, so the resistance state would be rather semiconducting than purely halfmetallic. The thermally activated behavior of resistivity governed by the Arrhenius model [50]

(1) ρ(T)=ρ0 eBT

can well describe the electrical transport mechanism for semiconducting and half-metallic materials, where $B=E_{a}/k$_B_. *E_a_* and *k* are activation energy and Boltzmann’s constant, respectively. The Arrhenius behavior for ρ(T) was reported before in Fe_3_O_4_ epitaxial films above the *T_V_* [51]. In addition, the ρ(T) for metallic Pt is fitted using the equation a + bT + cT^2^. Considering both layers (Pt and BPIO) as independent channels for charge transport with their resistances connected parallel to each other, the ρ(T) curve above the *T_V_* is fitted well with the expression:

(2) ρFe3O4Pt=ρFe3O4×ρPtρFe3O4+ρPt= (ρ0 eBT)×(a+bT+cT2)(ρ0 eBT)+(a+bT+cT2)

as shown in Figure 4b. Below the *T_V_*, variable range hopping (VRH) transport is the dominating mechanism for charge transport as the BPIO layer transforms to a semiconducting phase with higher resistance (where the Fe_3_O_4_ phase transforms from halfmetallic to semiconducting state with comparatively higher resistance [51], whereas the α-Fe_2_O_3_ phase remains insulating throughout the measured temperature range). In order to understand the nature of the VRH mechanism in the BPIO layer, we attempted to fit our ρ(T) data below the *T_V_* with both Mott’s 3D model [52],

(3) $$\rho =A\cdot e^{{\left(\frac{Q}{kT}\right)}^{\frac{1}{4}}},$$

as well as Efros-Shklovskii (ES) model [53],

(4) $$\rho =A\cdot e^{{\left(\frac{Q}{kT}\right)}^{\frac{1}{2}}}.$$

By comparing the Mott’s and ES fitting data, as indicated in Figure 4c, the best fit was obtained for Mott’s formula for a three-dimensional system [26]. However, the deviation from the VRH model below 40 K indicates the presence of additional mechanisms. Deviation from the VRH mechanism below ~60 K was observed before on an epitaxial Fe_3_O_4_ film [51]. Based on pure Pt’s temperature-dependent resistance (Figure S3), we confirm that neither Pt nor pure BPIO is responsible for the dip-like feature in ρ(*T*) since the resistivity for individual Pt and BPIO continuously decreases and increases with lowering temperature, respectively. To elucidate the low temperature electrical conduction mechanism, elastic electron-electron (e-e) scattering, and Kondo-like scattering are considered [54,55,56]. The former mechanism happens in inhomogeneous materials when temperature is low enough such that electrons experience a Coulomb interaction. Hence, the quantum correction causes the resistivity minimum [57,58]. The e-e interaction has a T^1/2^ contribution towards the resistivity. Kondo-like scattering is due to the magnetic impurities and therefore ln(T) behavior is expected [59]. In addition to the elastic scattering processes, the inelastic scattering due to electron-phonon (e-ph) and electron-magnon (e-m) scattering should also be considered. Since, in this case, the magnon propagation of Fe_3_O_4_ is short relative to the thickness of the sample [60], the e-m scattering channel is not relevant here. Therefore, resistivity can be corrected as:

(5) $$\rho =\rho_{elastic}+\rho_{inelastic\;},$$

where $\rho_{inelastic}$~*T*^5^ for e-ph scattering. The correction of the resistivity can be written as: $\rho_{el-el}$ *T*^1/2^ + $\rho_{e-Ph}$ *T*^5^ for e-e interaction and $\rho_{K}ln\left(T\right)$ + $\rho_{e-Ph}\;T^{5}$ for Kondo-like scattering. However, two scenarios are fitted well as shown in the Figure S2. We combine the e-e scattering and Kondo-like scattering together as:

(6) $$\rho =\rho_{0}+\rho_{K}ln\left(T\right)+\rho_{el-el}T^{1/2}+\rho_{e-ph}\;T^{5}.$$

The result shown in Figure 4d indicates that the low temperature minimum, *T_min_*, behavior has contributions from both types of scattering. The reason for this behavior may originate from the diffusion of Fe into Pt causing magnetic impurities to become embedded inside Pt [33]. Since BPIO is more resistive at low temperatures, shunting current to the Pt layer could become more significant. As a result, the effect of a thin disordered interface could be more prominent at low temperatures. To compare the difference between Pt/BPIO and Pt/WS_2_/BPIO, the equation is also applied to Pt/WS_2_/BPIO. The fitting parameters are compared for both Pt/BPIO and Pt/WS_2_/BPIO. For Pt/BPIO, the parameters $\rho_{0}$, $\rho_{K}$, $\rho_{el-el}$, and $\rho_{e-ph}$ are determined to be 13.04 ± 0.006, −0.132 ± 0.012, 0.0555 ± 0.007, and 7.234 × 10^−10^ ± 2.910 × 10^−10^, respectively. For Pt/WS_2_/BPIO, values of $\rho_{0}$, $\rho_{K}$, $\rho_{el-el}$, and $\rho_{e-ph}$ are 15.145 ± 0.004, −0.148 ± 0.009, 0.059 ± 0.005, and 1.336 × 10^−9^ ± 1.840 × 10^−10^, respectively. These results indicate that in the temperature range around this minimum behavior in ρ(T), Pt/WS_2_/BPIO has slightly stronger e-p scattering, with one order difference between these two systems. However, the e-e interaction and Kondo-like scattering do not have a significant difference. Interestingly, the local minimum behavior due to the interface interdiffusion did not manifest in the magnetic measurements. The main reason is that the dilute-magnetic semiconducting interface has much smaller magnetic signal compared to the magnetic property of bulk BPIO.

### 3.5. Atomically Resolved Spin Polarized Density of State

**Figure 5:**
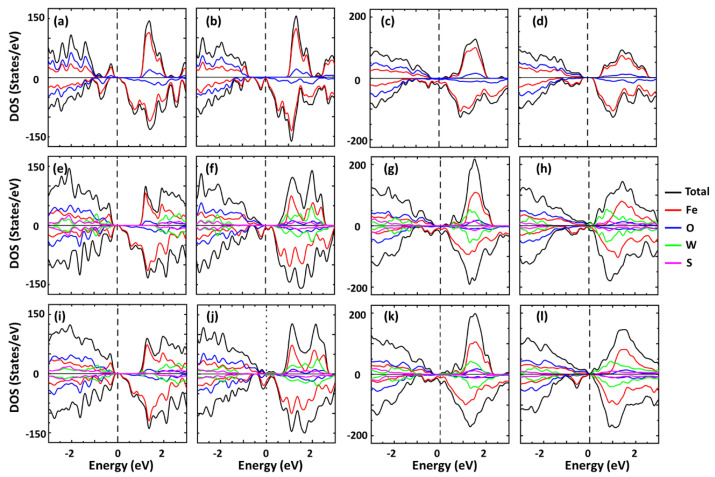
Total and atomically resolved density of states for a standalone Fe_3_O_4_ layer: (**a**) cubic−B, (**b**) cubic−AB, (**c**) monoclinic−B, (**d**) monoclinic−A Fe_3_O_4_ layer; and for Fe_3_O_4_/WS_2_ heterostructures: (**e**) cubic−B/WS_2_, (**f**) cubic−AB/WS_2_, (**g**) monoclinic−B/WS_2_, (**h**) monoclinic−A/WS_2_, (**i**) cubic−B/WS_2_ (V_S-S_), (**j**) cubic−AB/WS_2_ (V_S-S_), (**k**) monoclinic−B/WS_2_ (V_S−S_), (**l**) monoclinic−A/WS_2_ (V_S−S_). In all cases, the top two atomic layers of Fe_3_O_4_ are allowed to relax, while the bottom four layers are kept at their bulk positions (see Section 2).

The phenomenological model discussed above is further enhanced by the calculated electronic structure of WS_2_/Fe_3_O_4_ heterostructures from first principles. In Figure 5, we show the results for the total and atomically resolved spin polarized DOS for the various systems. It is noted that the surface termination and structure phase are important for the electronic properties of the magnetite. Thus, letting the top two atomic layers relax, while the bottom four layers are kept fixed at their bulk positions (see Figure 3), is a practical way to capture the specific surface termination within a given structural phase of the magnetite [44,45]. We find that the cubic-B Fe_3_O_4_ is a ferromagnetic semiconductor (Figure 5a), while the cubic-AB termination increases the energy gap for spin “up” carriers, and spin “down” carriers exhibit halfmetallic behavior (Figure 5b). On the other hand, the monoclinic-B Fe_3_O_4_ is a half metal (Figure 5c) and the monoclinic-A Fe_3_O_4_ is a ferromagnetic semiconductor (Figure 5d). The proximity of the WS_2_ changes the DOS to various degrees. For example, Figure 5e shows that the WS_2_/Fe_3_O_4_ heterostructure is a semiconductor whose energy gaps for spin “up” and “down” are similar to the standalone cubic-B Fe_3_O_4_ layer. The presence of defects in WS_2_ does not alter this behavior. On the other hand, the spin “up” energy gap for the cubic-AB Fe_3_O_4_/WS_2_ is reduced when compared to the cubic-AB Fe_3_O_4_, and the defective WS_2_ monolayer turns the heterostructure in spin-polarized metal due to the non-zero DOS at the Fermi level (Figure 5f). The monoclinic-B Fe_3_O_4_/ WS_2_ exhibits similar behavior as the Fe_3_O_4_ (Figure 5g) and the defects in WS_2_ (Figure 5k) reduce the gap of the spin “up” carriers of the heterostructure when compared with the monoclinic-B Fe_3_O_4_ layer. Interestingly, the transition metal dichalcogenide results in a metallic DOS for both types of spin polarization of the monoclinic-A/Fe_3_O_4_ heterostructure, but the S vacancies lead to a reduction of DOS at the Fermi level (Figure 5l). Our calculations further show that in all cases, hybridization between the d-orbital of Fe and W occurs in the highest conduction and lowest valence ranges. The effect is the largest for the monoclinic-A Fe_3_O_4_/WS_2_ heterostructure, especially for $d_{x^{2}-y^{2}}$ and $d_{z^{2}}$ state from Fe and W in the Fermi level region. The semiconducting nature of the Fe_3_O_4_ found in the calculations can be correlated with the FP measurement that is fitted by the Arrhenius equation and VRH mechanism.

### 3.6. Anomalous Hall Effect Measurement

**Figure 6:**
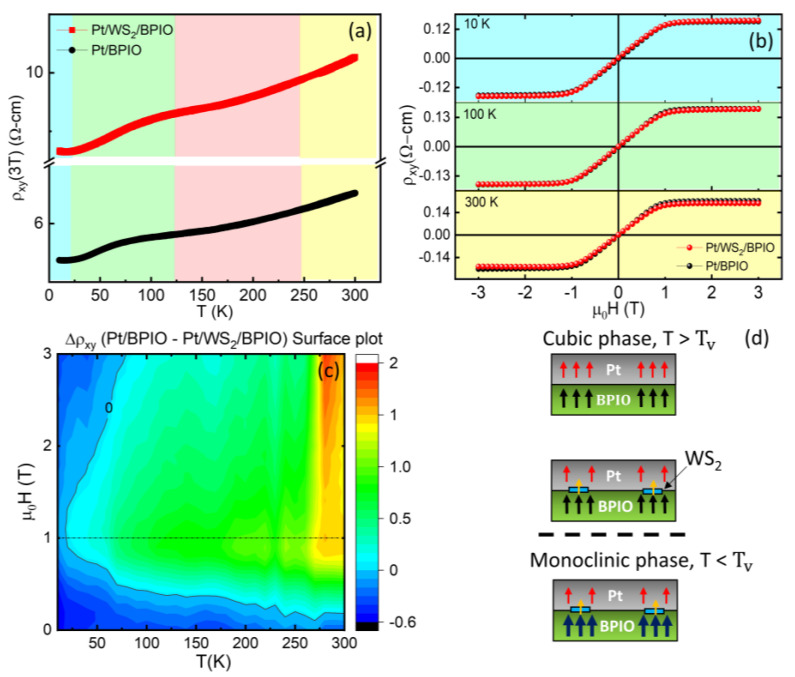
AHE measurements for Pt/BPIO and Pt/WS_2_/BPIO: (**a**) temperature−dependent *ρ_xy_* at 3 T and (**b**) *ρ_xy_* at selected temperatures of 10, 100, and 300 K. (**c**) 2D surface plot of the *ρ_xy_* difference between Pt/BPIO and Pt/WS_2_/BPIO. (**d**) Schematics showing possible differences in MPE between different Fe_3_O_4_ phases (cubic and monoclinic) and Pt, as well as the insertion of WS_2_. Red, black, and yellow arrows indicate Pt, WS_2_, and BPIO magnetization, respectively.

To further understand the interfacial effects, anomalous Hall effect (AHE) measurements were performed for Pt/BPIO and Pt/WS_2_/BPIO samples. When measuring resistivity of the films, the total resistivity is both contributed from ordinary Hall effect (OHE) and AHE. Hence, it can be written as:

(7) $$\rho_{xy}=R_{0}H_{z}+R_{S}M_{z},$$

where *R_0_*, *R_s_*, *H_z_* and *M_z_* are the ordinary Hall coefficient, anomalous Hall coefficient, magnetic field along out of plane direction, and magnetization, respectively. The temperature dependence of *ρ_xy_* at 3 T was measured, and a gradual decrease of *ρ_xy_*(*T*) is observed (Figure 6a). Close to the saturating resistivity (*ρ_sat_*) of the samples, the main contribution comes from AHE. When the temperature decreases, Fe_3_O_4_ becomes more and more insulating, hence the spin Hall Anomalous Hall resistivity (*ρ_SH-AHE_*) is more dominant. The *ρ_SH-AHE_* is dominated by the imaginary part of spin mixing conductance [61], which has a relatively small anomalous Hall signal compared to the traditional AHE. In the AHE measurement, *T_V_* and *T_min_* can still be observed in both the samples. However, for *T_min_*, both samples significantly reduce minimum point (14–16 K). This reason is possibly due to the application of a strong field (in this case, 3T), weakening the Kondo-like behavior. Figure 6b shows *ρ_xy_* measured at three temperatures: 10, 100, and 300 K for Pt/BPIO and Pt/WS_2_/BPIO samples. When reaching *ρ_sat_*, slightly larger *ρ_xy_* for Pt/BPIO at 300 K is observed compared to Pt/WS_2_/BPIO. With continuously decreasing temperature, Pt/WS_2_/BPIO slightly increases *ρ_xy_* and surpasses Pt/BPIO when temperature is below ~100 K. Figure 6c depicts the 2D surface plot of *ρ_AHE_* difference between Pt/BPIO and Pt/WS_2_/BPIO. Around room temperature, the Pt/BPIO has significantly higher *ρ_xy_* than Pt/WS_2_/BPIO (see Figure 6b). Pt and BPIO exhibit the MPE at high temperature since Pt is closer to a Stoner instability, which means that Pt is easier to induce magnetization. It has been reported in XMCD measurements that Pt and Fe-based complex oxides, such as Tm_3_Fe_5_O_12,_ has a higher onset MPE temperature [62], and therefore Pt atomic layers close to the interface between Pt and BPIO can be easily proximitized (the top part of Figure 6d). However, Pt/WS_2_/BPIO has local WS_2_ flakes which increase the distance between Pt and BPIO and block the MPE between Fe_3_O_4_ and Pt (the middle panel of Figure 6d). Note here that we simply consider the MPE between Fe_3_O_4_ phase and Pt. Considering the high temperature Fe_3_O_4_ cubic phase, the calculations show that there is no enhancement in the magnetization, and rather, M is reduced in the cubic-AB/WS_2_ with defects in the TMD monolayer. The results of calculated high temperature cubic phase indicate not only WS_2_ blocks the MPE between Pt and Fe_3_O_4_ but also AB-cubic/WS_2_ with vacancy reduces the magnetization. Moreover, the AB-terminated cubic magnetite shows a halfmetallic behavior (Figure 5b–j) which explains the conducting nature above *T_V_*. As a result, Pt/BPIO has the higher magnetization compared to Pt/WS_2_/BPIO. At temperatures below *T_V_*, $\rho_{xy}$ shows slightly higher values in Pt/BPIO, which is opposite to the behavior above *T_V_*, and it is consistent with the negative values in the 2D surface plot. Connecting with the simulations, we see from Table 1, that the monoclinic-A/WS_2_ exhibits enhancement in the magnetization compared to the bare monoclinic-A case. Although the total M reduces after considering vacancy inside WS_2_, it still remains higher than the monoclinic-A one. Considering the B termination of the monoclinic phase, the magnetization enhances only after the insertion of vacancy. To further confirm the possibility of magnetization enhancement, we compare the results of DOS in Figure 5. Before any insertion of WS_2_, the B termination already has a halfmetallic behavior. However, A termination conveys a semiconducting property at monoclinic phase, similar to the case of low temperature Fe_3_O_4_ semiconducting/insulating behaviors. This explains the low temperature AHE enhancement behavior that is contributed from the monoclinic-A termination. The choice of the different surface terminations for the two temperature regimes (above and below the *T_V_*) can be reconciled with the polycrystalline characteristic of the BPIO film. According to the discussion above, the results of the magnetization enhancement can be attributed to the orbital hybridization between Fe_3_O_4_ and WS_2_, as shown in the bottom panel of Figure 6d. In order to distinguish the enhancement of the overall magnetization in the system, the BPIO magnetization in the monoclinic phase changes from the thinner black arrows towards the thicker dark blue arrows. In addition, the magnetization of Pt/WS_2_/BPIO enhances with further reducing temperature. It has been previously shown that in a MoS_2_/YIG system, valley polarization increases with lowering temperature [5]. This can be attributed to the suppression of thermal fluctuation with lowering temperature. In the low field region (<1T), Pt/WS_2_/BPIO has higher values of *ρ_xy_* among all temperature regions of interest. Since OHE is inversely proportional to the carrier concentration [63], local WS_2_ flakes might lower the total carrier concentration owning to Pt being much more conductive. Consistent with the behavior of semiconducting materials, carrier concentration of WS_2_ drops with decreasing temperature. Therefore, the difference in the resistivity at low field increases with decreasing temperature. In addition, it is interesting to note that the Pt/WS_2_/BPIO magnetization is significantly enhanced with increasing field (>1T), which can be attributed to the change in spin alignment (from AFM to FM) within the Fe_2_O_3_ phase (Figure 2b), besides the Fe_3_O_4_ phase.

## 4. Conclusions

In conclusion, we have explored the properties of biphase BPIO (Fe_3_O_4_ + α-Fe_2_O_3_) and utilized the phase transitions in BPIO to probe the interfacial magnetic phenomena of 2D-TMD/FM heterostructures. From the experiments, the low temperature transition temperature found in the four-probe measurements was attributed to e-e scattering and Kondo-like behaviors based on the fitting results. A detailed analysis of the interfacial properties of Pt/WS_2_/BPIO shows the anomalous Hall resistivity enhancement at low temperatures, below the Verwey transition temperature. The enhancement of the resistivity in AHE measurements for Pt/WS_2_/BPIO comes from the orbital hybridization between the monoclinic-A Fe_3_O_4_ and WS_2_, which are confirmed by DFT calculations. In addition, the atomically resolved DOS shows the monoclinic-A terminated Fe_3_O_4_ interface with WS_2_ significantly changes the semiconducting behavior of the monoclinic-A Fe_3_O_4_ towards the metallic behavior, which can be attributed to the enhancement of the anomalous Hall resistivity. Both experiments and calculations confirm that the insertion of monolayer WS_2_ leads to the magnetization enhancement of the BPIO/WS_2_/Pt system. In future research, lower thicknesses of Fe_3_O_4_, as well as other conducting/insulating magnetic substrates may be used to fully explore and understand the MPE and spin transport across non-van der Waals magnet/van der Waals TMD interfaces. In addition to non-magnetic TMDs, magnetic TMD monolayers achieved recently via magnetic doping, also known as 2D diluted magnetic semiconductors [64,65], may be interfaced with non-van der Waals magnets to create heterostructures with optically controlled magnetic properties for opto-spin-caloritronics [4,66].

## Figures and Tables

**Table 1:** Basic properties of the considered heterostructures and standalone Fe_3_O_4_ layers with cubic-B, cubic-AB, monoclinic-B, and monoclinic-B surface terminations. The interlayer distance d (Å), binding energy $E_{b}=\left(E_{HST}-\underset{i}{\sum }E_{i}^{layer}\right)/N$ ($E_{HST}$ is the total energy of the heterostructure, $E_{i}^{layer}$ is the total energy of the ith isolated layer, N is the number of atoms in the supercell), average magnetic moments for the inequivalent atoms of Fe_3_O_4_ $m_{Fe_{A}},\;m_{Fe_{B}},\;m_{O}$, the total magnetization per atom of the WS_2_ monolayer $M_{WS_{2}}$, and total magnetization per atom of the entire structure M (all magnetic properties are in $\left(\mu_{B}\right)$ units) are shown.

	Interlayer Distance *d* (Å)	$\mathrm{Binding}\;\mathrm{Energy}\;E_{b}(\mathrm{meV})$	$m_{Fe_{A}}\;$ $\left(\mu_{B}\right)$	$m_{Fe_{B}}\;$ $\left(\mu_{B}\right)$	$m_{O}\;$ $\left(\mu_{B}\right)$	$M_{WS_{2}}\;$ $\left(\mu_{B}\right)$	M $\left(\mu_{B}\right)$
Cubic-B	-	-	−3.865	3.983	0.118	-	0.667
Cubic-B/WS_2_	2.521	−21.967	−3.862	3.988	0.114	0.003	0.667
Cubic-B/WS_2_ (VS-S)	2.505	−22.981	−3.862	3.986	0.115	0.003	0.667
Cubic-AB	-	-	−3.875	3.983	0.118	-	0.667
Cubic-AB/WS_2_	2.642	−34.880	−3.871	3.994	0.112	0.006	0.667
Cubic-AB/WS_2_ (V_S-S_)	2.537	−35.288	−3.869	4.000	0.113	0.003	0.666
Monoclinic-B	-	-	−3.907	4.007	0.139	-	0.667
Monoclinic-B/WS_2_	2.822	−20.322	−3.886	3.992	0.134	0.003	0.667
Monoclinic-B/WS_2_ (V_S-S_)	2.780	−17.715	−3.887	3.997	0.136	0.002	0.668
Monoclinic-A	-	-	−3.887	3.971	0.129	-	0.667
Monoclinic-A/WS_2_	2.207	−30.058	−3.874	3.977	0.132	0.00006	0.672
Monoclinic-A/WS_2_ (V_S-S_)	1.958	−27.378	−3.867	3.986	0.127	−0.001	0.671

## Data Availability

The research data will be available upon request.

## Supplementary Materials

The following supporting information can be downloaded at: https://www.mdpi.com/article/10.3390/nano13040771/s1, Figure S1: Schematic diagram of the wet transfer process; Figure S2: Low temperature resistivity behaviors for Pt/BPIO and Pt/WS_2_/BPIO. Figure S3: Temperature-dependent resistance measurement of pure Pt.
