# Supplementary material for: Enhanced Magnetism and Anomalous Hall Transport through Two-Dimensional Tungsten Disulfide Interfaces
Source: Nanomaterials (Basel). 2023 Feb 18;13(4):771. doi: 10.3390/nano13040771 (PMC9967397; doi:10.3390/nano13040771)
Supplement: Supplementary file 1 [file nanomaterials-13-00771-s001.zip › nanomaterials-2223069-supplementary.pdf]

Supporting information

Enhanced Magnetism and Anomalous Hall Transport through Two-dimensional Tungsten Disulfide Interfaces

Chang-Ming Hung[1], Diem Thi-Xuan Dang[1], Amit Chanda[1], Derick Detellem[1], Noha Alzahrani[1], Nalaka A Kapuruge[1], Yen T.H. Pham[1], Mingzu Liu[2], Da Zhou[2], Humberto R Gutierrez[1], Darío A. Arena[1], Mauricio Terrones[2], Sarath Witanachchi[1], Lilia M. Woods[1,*], Hariharan Srikanth[1,*], and Manh-Huong Phan[1,*]

[1] Department of Physics, University of South Florida, Tampa, Florida 33620, USA

[2] Department of Physics, The Pennsylvania State University, University Park, PA 16802, USA

*Corresponding authors: phanm@usf.edu (M.H.P), lmwoods@usf.edu (L.M.W), sharihar@usf.edu (H.S)

S1. Wet transfer process

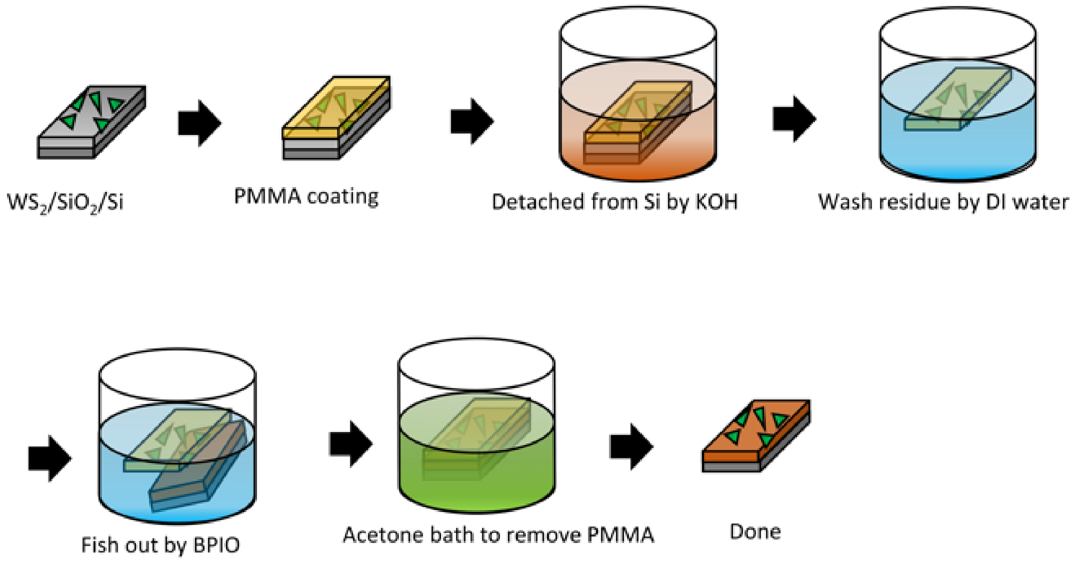

Figure S1. Schematic diagram of the wet transfer process.

S2. Pt/BPIO fitted by electron-electron interaction

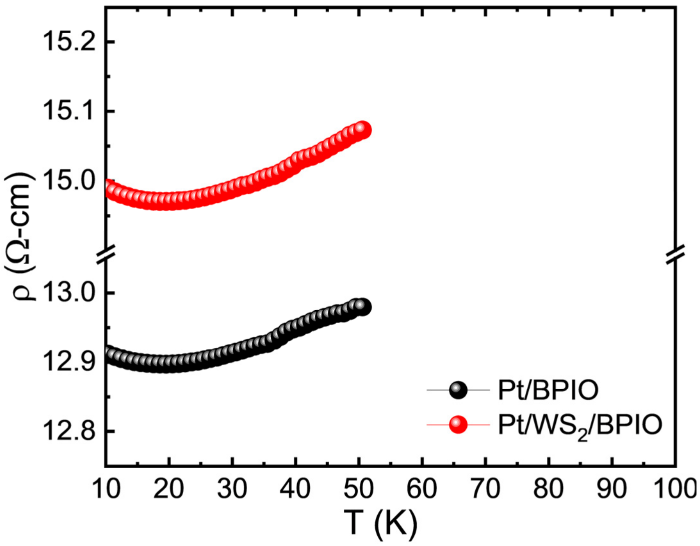

Figure S2. Low temperature resistivity behaviors for Pt/BPIO and $Pt/WS_2/BPIO$.

S3. Temperature-dependent resistance measurement of pure Pt

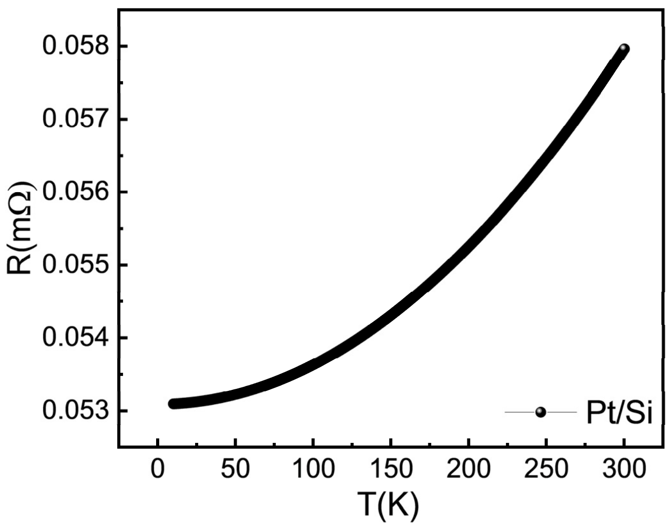

Figure S3. Temperature dependence of resistance of pure Pt
